# PDBe-KB: a community-driven resource for structural and functional annotations

**DOI:** 10.1093/nar/gkz853

**Published:** 2019-10-04

**Authors:** PDBe-KB consortium, Mihaly Varadi, John Berrisford, Mandar Deshpande, Sreenath S Nair, Aleksandras Gutmanas, David Armstrong, Lukas Pravda, Bissan Al-Lazikani, Stephen Anyango, Geoffrey J Barton, Karel Berka, Tom Blundell, Neera Borkakoti, Jose Dana, Sayoni Das, Sucharita Dey, Patrizio Di Micco, Franca Fraternali, Toby Gibson, Manuela Helmer-Citterich, David Hoksza, Liang-Chin Huang, Rishabh Jain, Harry Jubb, Christos Kannas, Natarajan Kannan, Jaroslav Koca, Radoslav Krivak, Manjeet Kumar, Emmanuel D Levy, F Madeira, M S Madhusudhan, Henry J Martell, Stuart MacGowan, Jake E McGreig, Saqib Mir, Abhik Mukhopadhyay, Luca Parca, Typhaine Paysan-Lafosse, Leandro Radusky, Antonio Ribeiro, Luis Serrano, Ian Sillitoe, Gulzar Singh, Petr Skoda, Radka Svobodova, Jonathan Tyzack, Alfonso Valencia, Eloy Villasclaras Fernandez, Wim Vranken, Mark Wass, Janet Thornton, Michael Sternberg, Christine Orengo, Sameer Velankar

**Affiliations:** 1 European Molecular Biology Laboratory, European Bioinformatics Institute (EMBL-EBI), Wellcome Genome Campus, Hinxton, UK; 2 Cancer Research UK Cancer Therapeutics Unit, Division of Cancer Therapeutics, The Institute of Cancer Research, London, UK; 3 School of Life Sciences, University of Dundee, Dundee, UK; 4 Department of Physical Chemistry, Palacky University, Olomouc; 5 University of Cambridge, Cambridge, UK; 6 Institute of Structural and Molecular Biology, University College London, Gower Street, London, WC1E 6BT, UK; 7 Weizmann Institute of Science, Rehovot, Israel; 8 Randall Centre for Cell & Molecular Biophysics, King's College London, London, UK; 9 European Molecular Biology Laboratory, Heidelberg, Germany; 10 Centre for Molecular Bioinformatics, Department of Biology, University of Rome Tor Vergata, Via della Ricerca Scientifica snc, 00133 Rome, Italy; 11 Charles University, Prague, Czech Republic; 12 Institute of Bioinformatics, University of Georgia, Athens, GA 30602, USA; 13 Cresset, Cambridgeshire, UK; 14 CEITEC, Central European Institute of Technology, Masaryk University, Brno, Czech Republic; 15 Indian Institute of Science Education and Research, Pune 411008, India; 16 University of Kent, Canterbury, Kent, CT2 7NJ, UK; 17 Centre for Genomic Regulation (CRG), Barcelona, Spain; 18 Barcelona Supercomputing Center, Barcelona, Spain; 19 Vrije Universiteit Brussel, Brussels, Belgium; 20 Imperial College London, London, UK; 21 Luxembourg Centre for Systems Biomedicine (LCSB), University of Luxembourg, Belvaux, Luxembourg; 22 National Centre for Biomolecular Research, Faculty of Science, Brno, Czech Republic

## Abstract

The Protein Data Bank in Europe-Knowledge Base (PDBe-KB, https://pdbe-kb.org) is a community-driven, collaborative resource for literature-derived, manually curated and computationally predicted structural and functional annotations of macromolecular structure data, contained in the Protein Data Bank (PDB). The goal of PDBe-KB is two-fold: (i) to increase the visibility and reduce the fragmentation of annotations contributed by specialist data resources, and to make these data more findable, accessible, interoperable and reusable (FAIR) and (ii) to place macromolecular structure data in their biological context, thus facilitating their use by the broader scientific community in fundamental and applied research. Here, we describe the guidelines of this collaborative effort, the current status of contributed data, and the PDBe-KB infrastructure, which includes the data exchange format, the deposition system for added value annotations, the distributable database containing the assembled data, and programmatic access endpoints. We also describe a series of novel web-pages—the PDBe-KB aggregated views of structure data—which combine information on macromolecular structures from many PDB entries. We have recently released the first set of pages in this series, which provide an overview of available structural and functional information for a protein of interest, referenced by a UniProtKB accession.

## INTRODUCTION

Since 1971, experimentally determined 3D structures have been deposited to the Protein Data Bank (PDB)—the single global archive for macromolecular structures (1). As of August 2019, the PDB contains more than 150 000 entries, referencing over 47 500 unique protein sequences in the Universal Protein Resource (UniProt) (2), with ∼12 000 new PDB structures added each year. The continuous improvement of experimental methods drives the expansion of the protein structural space covered by known structures. Ultimately, the goal of structure determination is to gain insights into the function of macromolecules (3), and to advance this goal, it is essential to place structural data in a biological context (4).

The wealth of structural data from the PDB is utilised by hundreds of data resources and scientific software. Many of these resources add valuable annotations and thereby, enhance the biological context of macromolecular structures. Such annotations include catalytic sites (5), ligand binding sites (6–8), molecular channels (9), post-translational modification sites (10,11) and other functional sites (12–14), context-dependent roles of small molecules (15,16), effects of genetic variability or mutations (17,18), dynamical properties and flexibility of biopolymer chains (19) and other biophysical parameters (20,21). Currently, the impact of these valuable annotations is limited by the following three factors: (i) the data is fragmented over a large number of distinct resources, each with its own data structure, formats and access mechanisms, making it difficult to compare or aggregate even similar types of annotations; (ii) many of these specialist resources typically reach only a comparatively small section of the scientific community; (iii) even expert users may not be aware of the full extent of the expanding ecosystem of these resources (22).

**Figure 1.**
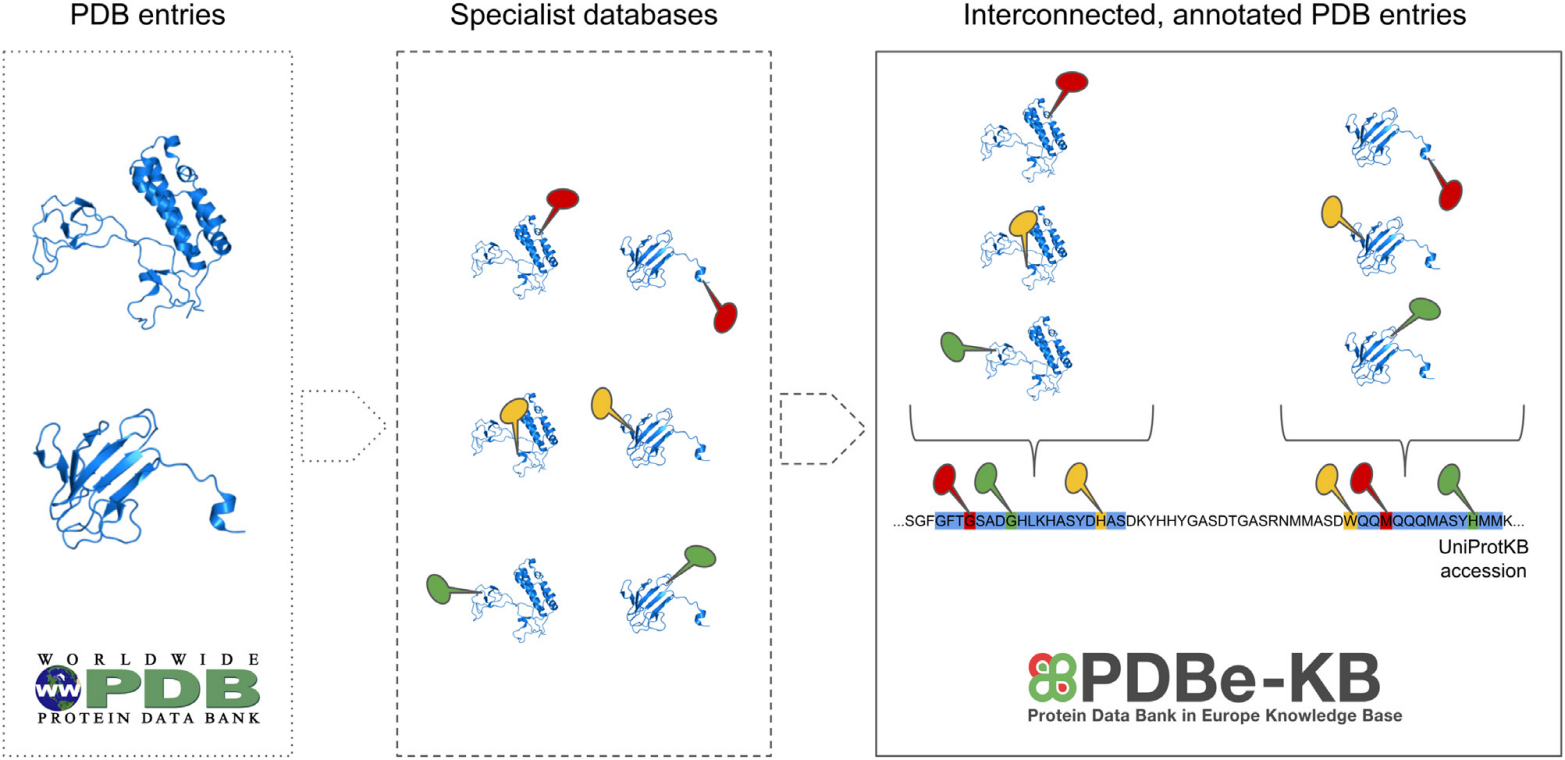
Traditionally, a PDB entry represents structures based on a single set of experiments, where each structure may represent only a segment of the full-length protein. However, PDB entries that describe the structure of the same protein are not interconnected. Furthermore, there is a rich ecosystem of resources and scientific software providing added value annotations based on the structures archived in the PDB, and when combined, these annotations provide evidence for the biological context of the protein. Therefore, the aim of PDBe-KB is to integrate these annotations and interconnect the various PDB entries in order to provide comprehensive, aggregated views of biologically meaningful entities, such as full-length proteins.

In an effort to align the management of these valuable data with the FAIR principles of Findability, Accessibility, Interoperability and Reusability (23), we have launched in 2018 the Protein Data Bank in Europe-Knowledge Base (PDBe-KB, pdbe-kb.org), a community-driven, collaborative resource, whose aim is to place macromolecular structures in their biological context by bringing together the various resources providing pieces of this context (Figure 1).

To facilitate this data integration, PDBe-KB partners have defined a common data exchange schema and format (available at https://gitlab.ebi.ac.uk/pdbe-kb/funpdbe/funpdbe-schema) for functional annotations of PDB data. The schema focuses on the commonalities of the annotations, capturing the minimal required information that can describe them, and provides links to more comprehensive views of the data hosted by the contributing partners, allowing users to explore the complete data available at the specialist data resources. This arrangement ensures that PDBe-KB remains scalable and maintainable while also increasing the visibility of the partner resources, and thus enhancing the sustainability of the data.

In 2018, PDBe-KB launched a deposition system accepting annotations from partners, and an infrastructure to store and expose these data via a distributable database and an Application Programming Interface (API). Furthermore, in March 2019, PDBe-KB introduced a website for accessing and displaying the contributed annotations and PDB data. To date, 18 partner resources from 8 countries collectively contributed over 520 million manually curated or predicted residue-level annotations for PDB structures (Table 1).

**Table 1.** Partner resources contributing annotations to PDBe-KB

Partner resource (Reference)	Resource leader	Type of annotations	Number of PDB entries
COSPI-Depth (21)	M. S. Madhusudhan	Residue depth	141 097
P2rank (6)	D. Hoksza	Binding site predictions	138 892
Arpeggio (15)	T. Blundell	Ligand interactions	117 023
3DComplex (14)	E. D. Levy	Interaction interfaces	111 555
DynaMine (19)	W. Vranken	Backbone flexibility predictions	98 548
POPSCOMP (20)	F. Fraternali	Solvent accessibility	77 578
AKID (11)	M. Helmer-Citterich	Kinase-target predictor	41 492
ChannelsDB (9)	R. Svobodova	Molecular channels	25 351
CATH-FunSites (13)	C. Orengo	Functional site predictions	23 975
canSAR (7)	B. al-Lazikani	Druggable pocket predictions	17 804
FoldX (17)	L. Serrano	Energetic consequences of mutations	3778
ProKinO (10)	N. Kannan	Curated regulatory sites	3673
14–3-3-Pred (12)	G. Barton	Binding site predictions	1941
CaMKinet *(in preparation)*	M. Kumar	Curated PTM sites	1076
M-CSA (5)	J. Thornton	Curated catalytic sites	919
3DLigandSite (8)	M. Wass	Binding site predictions	910
Missense3D (18)	M. Sternberg	Mutations in Human Proteome	0*
MetalPDB (16)	A. Rosato	Curated metal binding sites	0*
ELM (24)	T. Gibson	Short linear motifs	0*

PDBe-KB integrates data contributed by partner resources who provide a wide array of functional and biophysical annotations for PDB structures.

* Transfer of annotations is in progress.

PDBe-KB is managed by the Protein Data Bank in Europe team (PDBe; pdbe.org) (25) at the European Bioinformatics Institute. PDBe-KB is also one of the activities of the ELIXIR 3DBioInfo community (26), which aims to further integrate annotations and bioinformatics data related to macromolecular structure and to act as a forum for structural bioinformatics resources and research teams. PDBe-KB partners have agreed resource governance guidelines (http://pdbe-kb.org/guidelines), whose key points are summarised below: (i) the data contributed to PDBe-KB is free from any restrictions on distribution and re-use; (ii) the partners are responsible for the quality of the data they contribute; (iii) protocols for data generation must be described in peer-reviewed publications; and (iv) in case of predicted/calculated annotations, the contributing partner commits to submit data updates at least once a year, i.e. to provide annotations for newer PDB entries and/or to update the existing annotations when the underlying algorithms change. Literature-based manually curated resources may contribute data updates less frequently. PDBe-KB convenes annual general meetings where these guidelines are reviewed, new/prospective members present their work, and the overall progress and plans are discussed. New areas of future research are also identified with regards to gaps in annotations, improved data visualization and benchmarking of computational methods that provide annotations.

## IMPLEMENTATION

**Figure 2.**
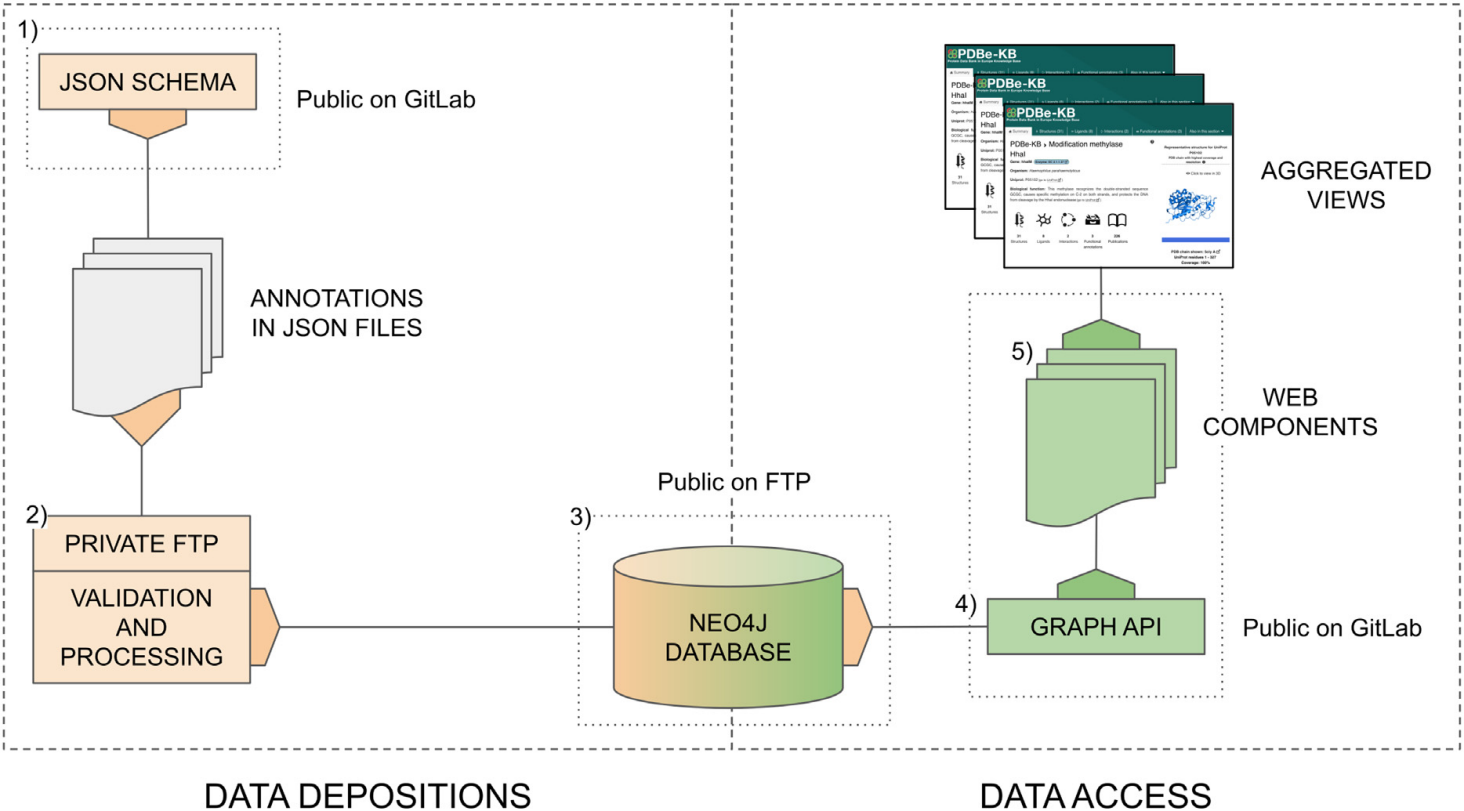
The infrastructure of PDBe-KB can be divided into data deposition and data access parts. Data deposition includes the data exchange format specification, the private FTP areas for depositors and the internal validation and processing pipeline hosted by PDBe. The data is integrated in a distributable graph database, and 50 public API endpoints serve data from it. These endpoints power all the reusable PDBe-KB web components. These web components are combined to create the aggregated views

The PDBe-KB infrastructure and workflow has two main parts (Deposition and Access) and can be divided into five components, described below and schematically depicted in Figure 2: (i) the data exchange JSON schema; (ii) the data deposition and validation system; (iii) the graph database that stores the aggregated data; (iv) the API enabling internal and external data access and (v) the reusable web components for data visualization.

### Data deposition

To facilitate data transfer and enhance interoperability, the consortium members have collaboratively designed a light-weight data exchange schema, available at https://gitlab.ebi.ac.uk/pdbe-kb/funpdbe/funpdbe-schema. The schema is a JSON specification and it defines the mandatory and optional fields required for deposition. The schema represents PDB entries hierarchically, with entry information at the root, and chains, and residues on subsequent levels. Residues may have multiple associated annotations, grouped together in ‘sites’ or ‘residue groups’ which provide labels for residues that belong together. For example, certain residues may together compose a druggable pocket (7), where each residue would have its distinct associated probability score, yet would be referenced to the same site, labelled ‘pocket 1’.

It is mandatory to provide residue-level annotations using the PDB author residue identifiers (i.e. the residue numbering provided by the original authors of the PDB entry) to map annotations to residues. In case of predicted annotations, the raw scores and confidence scores are also required. The depositors are encouraged to provide URLs linking to the original, complete datasets of which the deposited annotations are often a subset, although in some cases a generic URL to the resource or software can be provided.

The schema is maintained by the PDBe team and is updated according to the requirements of the consortium, tracking the changes in a changelog file. The repository of the schema also contains example JSON files that can serve as templates for prospective collaborators.

Deposition of annotations to PDBe-KB starts with discussions between new partners and PDBe, and after agreeing to the PDBe-KB guidelines, the prospective contributors convert their data to the PDBe-KB data exchange format described above. To facilitate the data conversion, a standalone Python package is provided to check if these files comply with the schema specification, as well as to perform all the consistency checks that the server-side deposition pipeline would perform. The package is available at https://gitlab.ebi.ac.uk/pdbe-kb/funpdbe/funpdbe-validator.

After the annotations are converted to compliant JSON files, they are transferred to private FTP areas provided by EMBL-EBI, and that serve as the authoritative sources for the annotations. The PDBe team runs an internal high-throughput validation and data processing pipeline weekly to perform all the checks and post-processing necessary for integrating the submitted annotations into the PDBe-KB graph database, described in the next section.

### Data access

One of the major advantages of building a knowledge base such as PDBe-KB is the co-location and interconnectedness of diverse types of information in a single environment. To capitalise on this advantage, all the annotations provided by PDBe-KB partners are integrated with the core PDBe data in a graph database powered by Neo4j (https://neo4j.com). Each PDB structure is represented as a tree in this graph, with the root being the PDB entry, connected to entities (unique molecules) and chains (instances of these molecules). Entities and chains are connected to residues. Where the mapping is available through the SIFTS data (27), PDB residues are connected to the corresponding residues in UniProtKB sequences. PDB residues (>150 million) are also linked with all the available PDBe-KB annotations, e.g. whether the given residue is part of a catalytic site or belongs to a macromolecular interaction interface.

Integrating PDB and PDBe-KB data as a graph allows the straightforward transfer of annotations from PDB entries to both directly mapped and to highly similar (90% identity) UniProtKB sequences and by extension to other PDB entries connected to those UniProt accessions. Weekly snapshots of the graph database index are made available on the PDBe FTP area at ftp://ftp.ebi.ac.uk/pub/databases/msd/graphdb/. The compressed snapshot required 102 Gb of storage (∼500 Gb uncompressed) as of August 2019.

To ensure consistent and robust access to all the PDBe-KB data stored in the graph database, the PDBe team has developed a REST API (implemented in the Flask framework for Python - https://palletsprojects.com/p/flask), which contains 50 public endpoints. Detailed documentation and examples for each endpoint are available at https://pdbe-kb.org/api. This API powers the new PDBe-KB web components and pages and is also used by PDBe entry pages, PDBe query system, and has already been integrated into some of the other EBI resources.

### Web components

**Figure 3.**
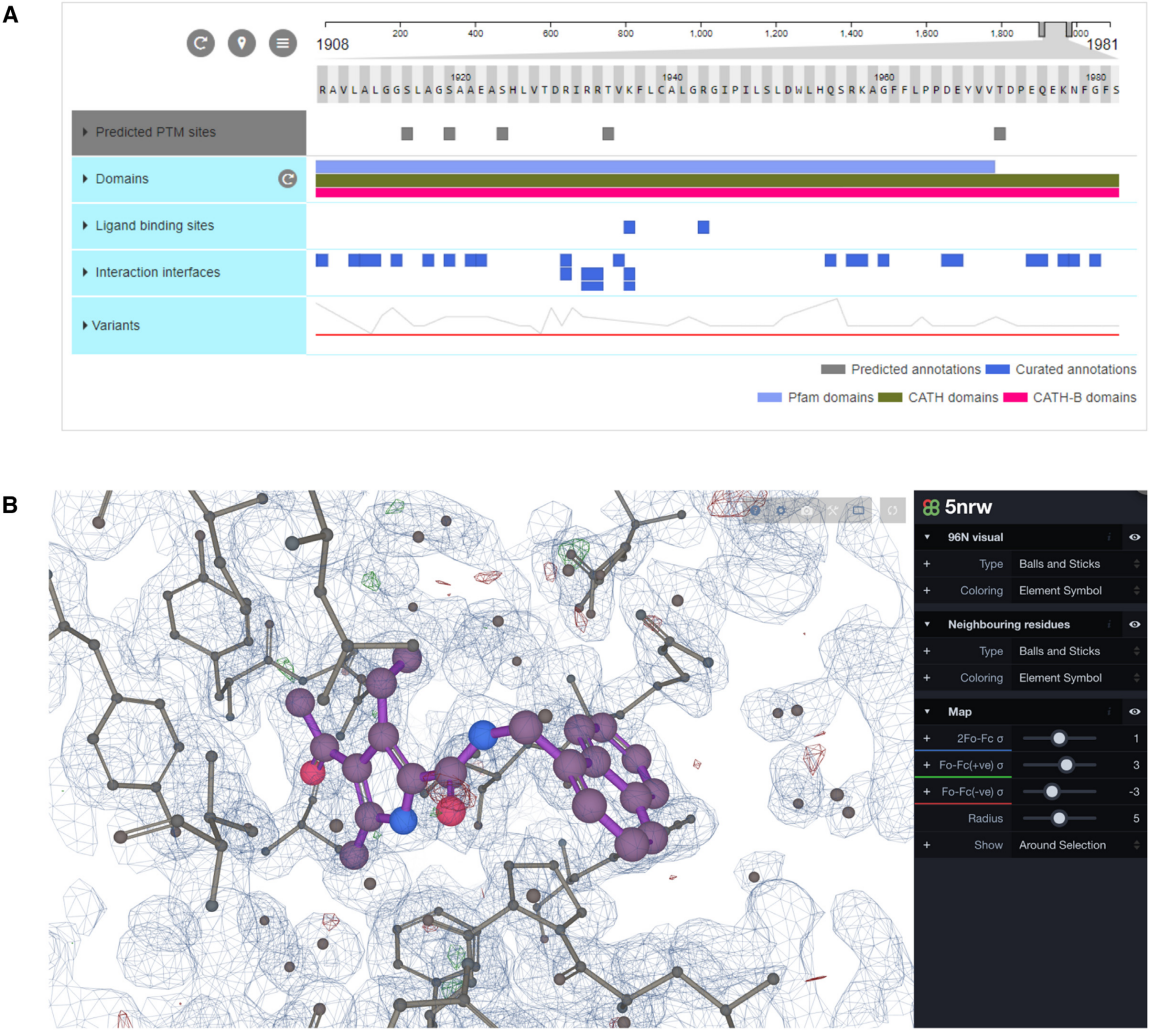
The aggregated protein views are built using several web components, the main components being ProtVista and LiteMol. ProtVista (**A**) is a sequence feature viewer co-developed by UniProt, PDBe and InterPro. It can be used to display residue-level information mapped to sequences. Our implementation of LiteMol (**B**) is a lightweight molecular viewer wrapped into a reusable web component that allows the visualisation of biological assemblies, complexes and ligand binding sites.

Clear and intuitive visualisation of structural data is crucial for conveying scientific information to the diverse user community. PDBe-KB presents the core PDB data and the biological context annotations on novel aggregated views for structural data. The first set of such views focus on full-length proteins (UniProtKB accessions). These views are assembled from modular, reusable and customisable web components which retrieve the data via a REST API (see above in the ‘Data access’ section). These components are implemented in the Angular 6+ framework (https://angular.io) and include textual and pictorial data representations and interactive visualizations. Specifically, to display PDBe-KB annotations we use the new version of the ProtVista sequence feature viewer (28) (Figure 3A), which is a visualization tool co-developed by UniProt (29), InterPro (30) and PDBe (25) (available at https://github.com/ebi-webcomponents/nightingale) and, in order to display annotations in 3D, we use the macromolecular viewer LiteMol (31) (Figure 3B, available at https://www.ebi.ac.uk/pdbe/pdb-component-library).

## AGGREGATED VIEWS OF PROTEINS

To display the functional annotations and structural data in a useful manner, we have developed a set of novel web pages (aggregated views) that provide an overview of all the structure data related to a full-length protein (UniProtKB accession), as opposed to the conventional pages that focus on single PDB entries. These novel views were designed following extensive gathering and analysis of user requirements and were launched in March 2019.

The aggregated views for proteins contain seven sections, which aim to answer specific scientific questions: (i) *summary*: how many PDB entries map to the UniProtKB accession? How many ligands and macromolecular partners are observed in the PDB interacting with the protein in question? How many types of annotations and how many publications are available? Which PDB entries contain representative structures of the protein, and what do these representative structures look like?; (ii) *structural coverage and domains*: what PDB entries are available for a given protein and their mapping onto to the UniProt sequence, and what domains are present in these entries?; (iii) *small molecules*: which small molecules are observed to either interact directly with the protein in question or are observed in PDB entries containing it?; (iv) *macromolecular interactions*: which macromolecules does the protein interact with according to the available data in PDB entries?; (v) *functional annotations*: what are the annotations deposited by PDBe-KB partner resources for the protein?; (vi) *similar proteins*: For proteins with structures available in the PDB, a list of similar proteins, with high sequence identity, from UniProtKB without any structure data and if there are no data in PDB for the protein of interest, are there proteins with high sequence identity that have structural data available in the PDB? and (vii) *publications*: which are the primary publications citing the PDB entries related to the protein? What reviews are available either based on PDB or UniProtKB accessions for this protein?

### Summary and structural overview

**Figure 4.**
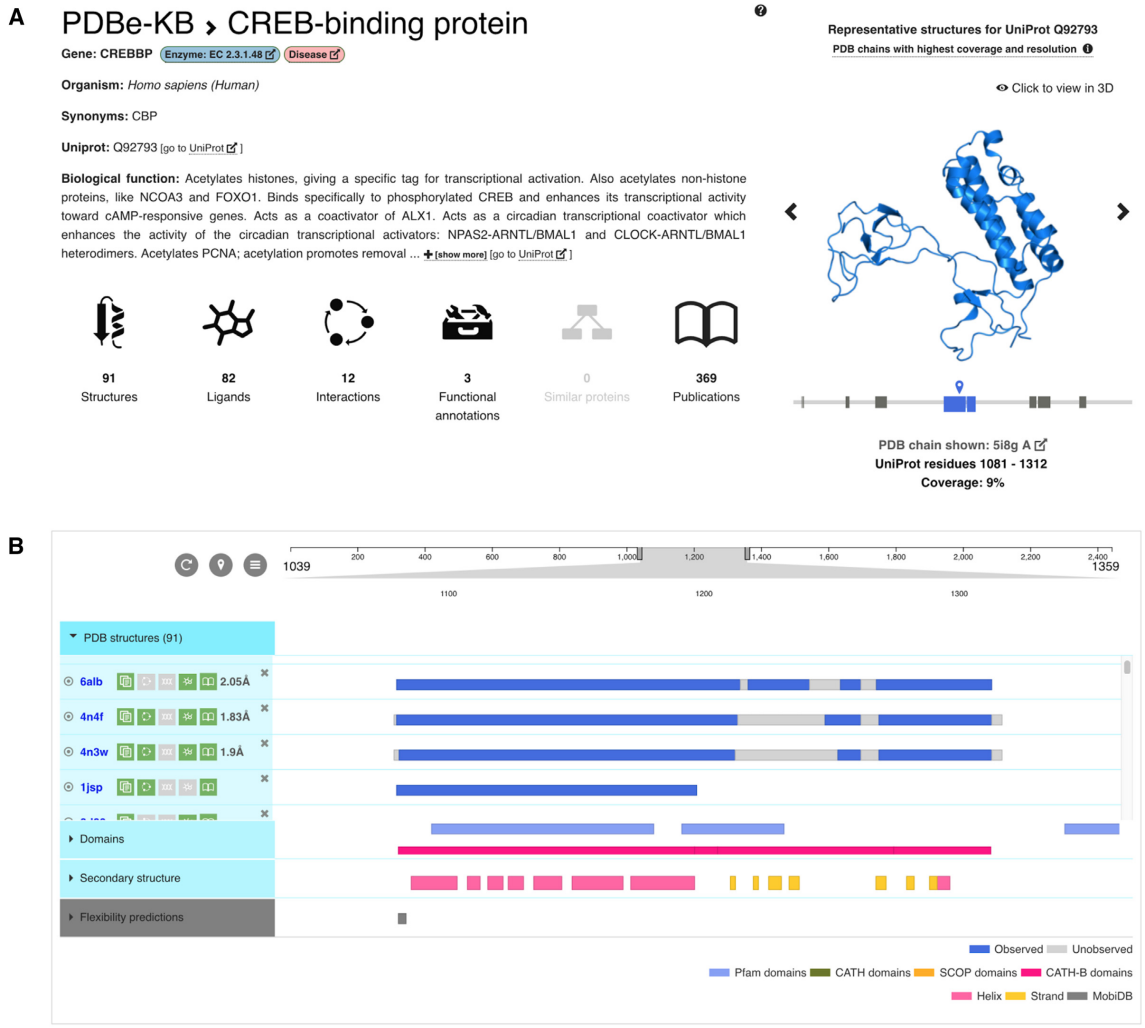
The first two sections of the aggregated views of proteins provide an overview of all the data available in PDB for a protein of interest. The view includes the number of interacting small-molecules, macromolecular interaction partners and functional annotations, as well as the number of publications related to the PDBs, mapped to the protein (**A**). It also offers visual help for identifying all the PDB entries that cover various segments of the protein, as well as showing representative non-overlapping structures both as static images and interactive 3D viewer (**B**).

The summary section provides an overview of all the available data using icons that also serve to navigate between the various sections (Figure 4A). Basic information such as the gene name, species and biological function of the protein is provided by the Proteins API (32). Known enzymes and proteins implicated in diseases are labelled accordingly. The section also provides a gallery of representative structures available for the various segments of the full-length protein. These representative PDB structures are selected based on sequence coverage, resolution and data quality (33). Structural studies of large proteins tend to yield multiple structures of shorter (often non-overlapping) segments of the primary sequence that are described in different PDB entries. This section aims to simplify the navigation between the various entries. Clicking on the images of any of the representative PDB structures launches an instance of the interactive 3D visualization tool LiteMol (31).

The Structures and Domains section provides an overview of all (i.e. not just the representative) PDB entries available for the protein and displays which region of the protein these PDB entries cover (Figure 4B). It also indicates which residues are modeled with atomic coordinates (i.e. observed), and which ones are not (i.e. unobserved), and allows identification of PDB entries containing particular domains from Pfam (34), CATH (35) and SCOP (36). Additionally, it displays the secondary structure content of the representative PDB chains and predictions of intrinsic disorder provided by MobiDB (37). All this information is displayed using an implementation of the ProtVista sequence feature viewer (28).

### Galleries and annotations

**Figure 5.**
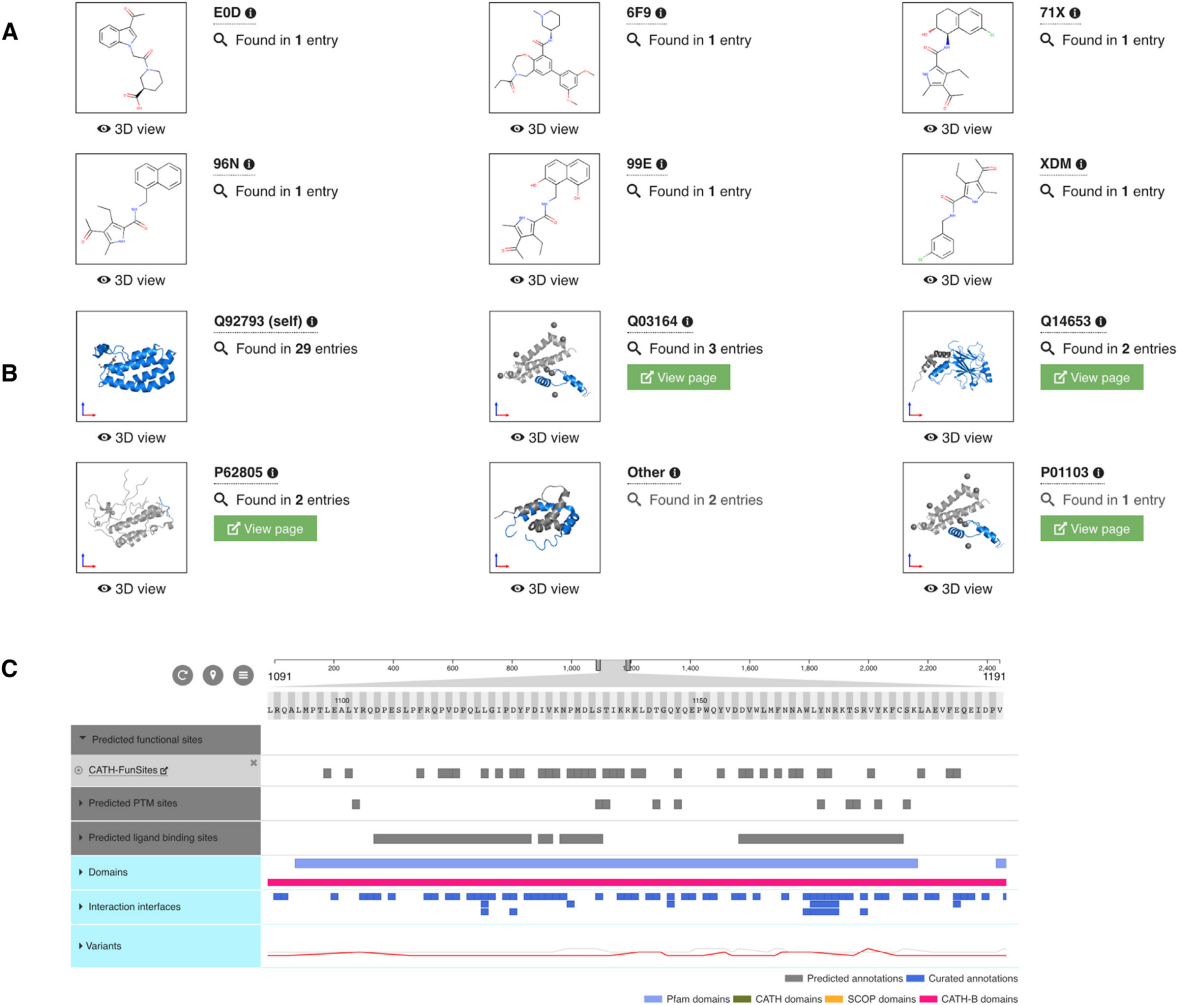
The aggregated protein views provide information on all the observed interactions between the protein of interest and ligands or macromolecular interaction partners. The gallery of ligands (**A**) and macromolecules (**B**) can be used to display their interactions in an interactive LiteMol instance, and to navigate to the corresponding PDBe and PDBe-KB entry pages to get more information about the partner molecules. Additionally, all the available functional annotations and biophysical parameters provided by PDBe-KB partner resources are being displayed using ProtVista (**C**).

The ligands and environments section displays a gallery of all the unique small molecules that are observed to be interacting with the protein of interest in any of the PDB entries (Figure 5A), as well as any small molecules observed in the same PDB entry as the protein of interest (i.e. not necessarily interacting directly). This gallery can be filtered using molecule names, three-letter molecule (chemical compound) identifiers and PDB identifiers, and clicking on each image displays the ligand and its environment in 3D using LiteMol (31). If the molecule acts as a cofactor (38), this is also indicated in this section.

Below the gallery, ProtVista is used to highlight all the residues of the protein that directly interact with each of the small molecules, as determined by the Arpeggio tool (15). Each row shows the residues interacting with a particular small molecule, while collapsing the category can provide an overview of all the binding sites, which can be used to find protein sequence patterns in protein–ligand interactions. This overview can thus help in differentiating between sporadic interaction sites of solvent molecules and functionally relevant binding pockets.

The macromolecular interactions section has a similar layout to the ligands and environments section and uses the same web components to display different data (Figure 5B). The gallery in this section shows all the macromolecular partners which are directly interacting with the protein of interest, in at least one of the PDB entries and highlights how many PDB entries this interaction is observed. It also provides links to the aggregated views for these partner proteins. The images can be clicked to display the complexes in 3D using LiteMol (31). Again, ProtVista is used to highlight all the residues that are directly interacting with the macromolecular partners, i.e. all the interaction interfaces are identified and listed.

The subset of the annotations provided by PDBe-KB partners are displayed in the additional annotations section of the first version of the aggregated views for proteins (Figure 5C). This section shows all the predicted and experimentally determined functional sites and biophysical parameters contributed by the partner resources listed in Table 1. Curated annotations are colored blue, while prediction-based annotations are colored grey, to allow clear visual distinction between the two categories. Additionally, all the available variation data from UniProt (29) are displayed. All these annotations (as well as any data displayed with ProtVista from any of the sections) can be downloaded in CSV and JSON formats.

### Similar proteins

The similar protein section shows all the UniProtKB accessions that are in the same UniRef90 cluster as the protein of interest, i.e. those proteins that share >90% sequence identity, further restricted to a minimum of 70% coverage by a single PDB structure (27). Further developing this section can help to transfer annotations and structural information from approximately 47 500 structurally characterised proteins (i.e. UniProtKB accessions represented in the PDB) to more than 2.2 million UniProtKB accessions reached via the UniRef90 clusters.

### Publications

The publication section provides links and summary information of publications that are either: (i) the primary publications of PDB entries related to the protein of interest; or (ii) reviews where these PDB entries are mentioned; or (iii) reviews that are specifically related to the UniProtKB accession of the protein of interest. These references can be filtered by keywords and exported in CSV and BibTeX format.

## CONCLUSIONS AND OUTLOOK

PDBe-KB is an open-access, collaborative and integrated resource managed by the PDBe team at EMBL-EBI. The goal of PDBe-KB is to place macromolecular structure data in their biological context, and therefore to help the broader scientific community to utilize these data in both fundamental and applied research and education, as well as to reduce the fragmentation of the valuable annotation data derived by many structural bioinformatics resources. PDBe-KB was established in 2018, and since then it has gathered contributions from 18 resources from 8 countries, providing over 520 million residue-level annotations. PDBe-KB recently became an activity of the ELIXIR 3DBioInfo community, which aims to bring together structural bioinformatics developers and scientists to work towards FAIRness of structural annotations data (23).

PDBe-KB aims to provide novel aggregated views of PDB data and their annotations, with the first set of such aggregated views, launched in March 2019, focusing on full-length proteins (i.e. UniProtKB accessions). We continue to work on displaying additional functional and structural annotations contributed by the PDBe-KB partner resources and by transferring annotations between proteins which are in the same UniRef90 cluster.

Recently, we have started designing the next set of aggregated views that will focus on small molecules in the PDB and will integrate the structural data from the PDB with the biological context annotations pertaining to small molecules. These small-molecule-centric views are expected to be released in the first half of 2020. Concurrently, in collaboration with the Complex Portal team (39) at EMBL-EBI we have started investigating the data and visualization requirements for creating aggregated views aimed at macromolecular complexes.

To further improve data accessibility, we packaged the PDBe-KB graph database and made it distributable so that the complete database can be installed and queried locally by the scientific community and industry partners alike.

We extend an invitation to explore the possibility of joining the PDBe-KB consortium to new partners who can fill the gaps in current PDBe-KB biological context annotations, in particular pertaining to nucleic acids, membrane proteins, peptides, antibodies and biophysical parameters derived from experimental characterization of small molecule binding sites and protein complexes.

In conclusion, PDBe-KB brings structural data and annotations closer to the broader scientific community by allowing convenient access to data that would have been otherwise available in fragments from many specialist data resources. PDBe-KB also serves as a platform that helps specialist data resources to increase their visibility and helps them to increase the FAIRness of their data.

## DATA AVAILABILITY

PDBe-KB is available at https://pdbe-kb.org and the protein aggregated views can be found at https://pdbe-kb.org/proteins. The data exchange format is available at https://gitlab.ebi.ac.uk/pdbe-kb/funpdbe/funpdbe-schema and the JSON validator that can help depositors check their data files can be found at https://gitlab.ebi.ac.uk/pdbe-kb/funpdbe/funpdbe-validator. The Neo4J graph database snapshots are available via FTP at ftp://ftp.ebi.ac.uk/pub/databases/msd/graphdb/. Finally, the API with 50 public API endpoints and their documentation is available at https://pdbe-kb.org/api.

## APPENDIX

**Current PDBe-KB Consortium Members with Affiliations**

Mihaly Varadi^1^, John Berrisford^1^, Mandar Deshpande^1^, Sreenath S. Nair^1^, Aleksandras Gutmanas^1^, David Armstrong^1^, Lukas Pravda^1^, Bissan Al-Lazikani^2^, Stephen Anyango^1^, Geoffrey J. Barton^3^, Karel Berka^4^, Tom Blundell^5^, Neera Borkakoti^1^, Jose Dana^1^, Sayoni Das^6^, Sucharita Dey^7^, Patrizio Di Micco^2^, Franca Fraternali^8^, Toby Gibson^9^, Manuela Helmer-Citterich^10^, David Hoksza^11,21^, Liang-Chin Huang^12^, Rishabh Jain^9^, Harry Jubb^13^, Christos Kannas^2^, Natarajan Kannan^12^, Jaroslav Koca^14,22^, Radoslav Krivak^11^, Manjeet Kumar^9^, Emmanuel D. Levy^7^, F. Madeira^1^, M. S. Madhusudhan^15^, Henry J. Martell^16^, Stuart MacGowan^3^, Jake E. McGreig^16^, Saqib Mir^1^, Abhik Mukhopadhyay^1^, Luca Parca^10^, Typhaine Paysan-Lafosse^1^, Leandro Radusky^17^, Antonio Ribeiro^1^, Luis Serrano^17^, Ian Sillitoe^6^, Gulzar Singh^15^, Petr Skoda^11^, Radka Svobodova^14,22^, Jonathan Tyzack^1^, Alfonso Valencia^18^, Eloy Villasclaras Fernandez^2^, Wim Vranken^19^, Mark Wass^16^, Janet Thornton^1^, Michael Sternberg^20^, Christine Orengo^6^, Sameer Velankar^1,*^

1 European Molecular Biology Laboratory, European Bioinformatics Institute (EMBL-EBI), Wellcome Genome Campus, Hinxton, UK

2 Cancer Research UK Cancer Therapeutics Unit, Division of Cancer Therapeutics, The Institute of Cancer Research, London, UK

3 School of Life Sciences, University of Dundee, Dundee, UK

4 Department of Physical Chemistry, Palacky University, Olomouc

5 University of Cambridge, Cambridge, UK

6 Institute of Structural and Molecular Biology, University College London, Gower Street, London, WC1E 6BT, UK

7 Weizmann Institute of Science, Rehovot, Israel

8 Randall Centre for Cell & Molecular Biophysics, King's College London, London, UK

9 European Molecular Biology Laboratory, Heidelberg, Germany

10 Centre for Molecular Bioinformatics, Department of Biology, University of Rome Tor Vergata, Via della Ricerca Scientifica snc, 00133 Rome, Italy

11 Charles University, Prague, Czech Republic

12 Institute of Bioinformatics, University of Georgia, Athens, GA 30602, USA

13 Cresset, Cambridgeshire, UK

14 CEITEC, Central European Institute of Technology, Masaryk University, Brno, Czech Republic

15 Indian Institute of Science Education and Research, Pune 411008, India

16 University of Kent, Canterbury, Kent, CT2 7NJ, UK

17 Centre for Genomic Regulation (CRG), Barcelona, Spain

18 Barcelona Supercomputing Center, Barcelona, Spain

19 Vrije Universiteit Brussel, Brussels, Belgium

20 Imperial College London, London, UK

21 Luxembourg Centre for Systems Biomedicine (LCSB), University of Luxembourg, Belvaux, Luxembourg

22 National Centre for Biomolecular Research, Faculty of Science, Brno, Czech Republic
